# Anti-vascular endothelial growth factor antibody attenuates inflammation and decreases mortality in an experimental model of severe sepsis

**DOI:** 10.1186/cc12742

**Published:** 2013-05-27

**Authors:** Su Jin Jeong, Sang Hoon Han, Chang Oh Kim, Jun Yong Choi, June Myung Kim

**Affiliations:** 1 Department of Internal Medicine and AIDS Research Institute, Yonsei University College of Medicine, Seoul, Republic of Korea

**Keywords:** sepsis, vascular endothelial growth factor, bevacizumab, anti-VEGF antibody

## Abstract

**Introduction:**

Severe sepsis is associated with an unacceptably high rate of mortality. Recent studies revealed elevated levels of vascular endothelial growth factor (VEGF), a potent angiogenic and vascular permeability factor, in patients with sepsis. There was also an association between VEGF levels and sepsis severity. Here we investigate the effects of an anti-VEGF antibody (Bevacizumab, Bev) in an experimental model of sepsis.

**Methods:**

Human umbilical vein endothelial cells (HUVECs), murine cecal ligation and puncture (CLP), and endotoxemia models of sepsis were used. HUVECs were treated with lipopolysaccharide (LPS) and/or Bev, harvested and cytokine mRNA levels determined using a semi-quantitative reverse transcription-polymerase chain reaction assay. The levels of inflammatory cytokine were also determined in HUVECs supernatants. In addition, the effects of Bev on mortality in the CLP and endotoxemia models of sepsis were evaluated.

**Results:**

Treatment with Bev and LPS significantly decreased the expression and the level of inflammatory cytokines in HUVECs relative to LPS alone. In CLP and endotoxemia models, survival benefits were evident in mice given 0.1 mg/kg of Bev relative to the CLP or LPS alone (*P *<0.001 and *P *= 0.028, respectively), and in 6 h post-treated mice relative to the CLP alone for the effect of different time of Bev (*P *= 0.033). In addition, Bev treatment inhibited LPS-induced vascular leak in the lung, spleen and kidney in the murine endotoxemia model (*P *<0.05).

**Conclusions:**

Anti-VEGF antibody may be a promising therapeutic agent due to its beneficial effects on the survival of sepsis by decreasing inflammatory responses and endothelial permeability.

## Introduction

Sepsis, the systemic inflammatory response to infection, is a leading cause of morbidity and mortality. Although the pathways that are activated during sepsis have been characterized extensively, much remains to be learned about the mechanisms underlying sepsis-induced organ failure. Thus far, efforts to block individual components of the inflammatory or coagulation pathways have had little impact on survival. Tumor necrosis factor-α (TNF-α) is one of the most potent pro-inflammatory cytokines identified in sepsis. However, a disconnect exists between hyper-acute experimental animal models and human sepsis illustrated by the failure of several clinical trials of anti-TNF-α monoclonal antibodies [1,2]. In addition, a recent randomized controlled trial using recombinant activated protein C (rhAPC) found no benefit, prompting withdrawal of this drug from the market [3]. Thus, mortality rates remain close to 25 to 30%. Clearly, future advances in therapy will be contingent upon an improved understanding of sepsis pathophysiology.

Vascular endothelial growth factor (VEGF) was first identified and characterized as a vascular permeability factor and then subsequently reported to promote proliferation, migration and survival of endothelial cells [4-6]. VEGF (also termed VEGF-A) is a member of a growing family of related proteins that include VEGF-B, -C, -D and placental growth factor (PIGF) [7]. VEGF binds to two transmembrane receptors, namely Flt-1 and Flk-1, whereas PIGF binds to Flt-1 alone. Within the vessel wall, Flk-1 is selectively expressed in the endothelium. Flt-1 is present on both endothelial cells and monocytes. In addition to its role in promoting endothelial permeability and proliferation, VEGF may contribute to inflammation and coagulation. For example, VEGF induces the expression of cellular adhesion molecules including E-selectin, intercellular adhesion molecule 1 (ICAM-1), and vascular cell adhesion molecule 1 (VCAM-1) in endothelial cells and promotes the adhesion of leukocytes [8,9]. Moreover, VEGF signaling up-regulates tissue factor mRNA, protein and procoagulant activity [10]. Recently, two independent studies reported an association between human sepsis/septic shock and elevated circulating levels of VEGF [11,12].

Bevacizumab (Bev) (Avastin; GeneTech, Inc., San Francisco, CA, USA) was the first humanized anti-VEGF neutralizing antibody approved by the Food and Drug Administration (FDA) for treatment of metastatic colon cancer [13]. Bev combined with chemotherapy has been used in clinical trials for several types of cancer [14]. Following the first intravitreal application of Bev in 2005 [15], its off-label use for exudative age-related macular degeneration is now widespread. However, no studies have evaluated the effectiveness of Bev in sepsis models. Therefore, we designed this study to test the hypothesis that VEGF plays a pathogenic role in mediating sepsis and that a humanized anti-VEGF neutralizing antibody, Bev, could be an effective therapeutic agent in a murine sepsis model. We determined whether Bev can attenuate lipopolysaccharide (LPS)-induced inflammation and improve survival. We also assessed whether Bev could affect expression and/or secretion of pro-inflammatory cytokines involved in LPS-toll like receptor (TLR)-4 signaling.

## Materials and methods

### Animal preparation and treatment

C57BL/6 mice were fed a standard laboratory diet with water *ad libitum *and treated according to the guidelines and regulations for the use and care of animals of Yonsei University, Seoul, Republic of Korea. Mice were seven to eight weeks of age and weighed 25 to 30 g at the start of the experiments. Animal experiments were reviewed and approved by the Institutional Animal Care and Use Committee of Yonsei University College of Medicine.

### Cecal ligation and puncture (CLP) procedures

CLP surgery was performed as described previously [16]. Briefly, the mice were anesthetized with an intraperitoneal (i.p.) injection of a 200-μL mixture of ketamine (9 mg/mL) and xylazine (1 mg/mL). The cecum was exteriorized through a 1 cm midline abdominal incision and then ligated distal to the ileocecal junction using 5.0 monofilament. Greater than 75% of the cecum was ligated. The antimesenteric side of the cecum was punctured bilaterally with a 23-gauge needle. A small amount of luminal contents was expressed through both puncture sites to ensure patency. The cecum was returned to the abdominal cavity, and the fascia and skin incisions were closed with 6.0 monofilament and surgical staples, respectively. Sham-operated mice underwent identical procedures, but without ligation and puncture of the cecum. Topical 1% lidocaine and bacitracin were applied to the surgical site post-operatively. All animals received a single intramuscular injection of trovafloxacin (Pfizer, New York, NY, USA) at a dose of 20 mg/kg and subcutaneous fluid resuscitation with 1.0 mL normal saline immediately post-surgery.

### Experimental design of murine CLP and endotoxemia models

The study was performed in two murine models of sepsis. First, we looked at the effect of different doses of Bev on mortality in the CLP-induced sepsis and endotoxemia mouse models. In the CLP model, these four groups consisted of the CLP-only group (*n *= 13) in which only CLP was performed, the CLP with 0.1 mg/kg Bev group (*n *= 8), the CLP with 1.0 mg/kg Bev group (*n *= 8) and a sham-surgery-only group (negative control, *n *= 5). The CLP-only group received only normal saline 1 h before CLP surgery; and Bev was administered 1 h before CLP surgery. The sham surgery group also received normal saline at 1 h before sham surgery. CLP has the disadvantages of variable severity due to differences in experimental procedures. Therefore, compared to the other groups, a larger number of mice were assigned to the CLP-only group. For the endotoxemia model, mice received i.p. injections of LPS (22 mg/kg weight) from *E. coli *serotype 0111:B4 (Sigma-Aldrich, St. Louis, MO, USA). The four mouse groups consisted of the LPS-only group (*n *= 5), the LPS with 0.1 mg/kg Bev group (*n *= 10), the LPS with 1.0 mg/kg Bev group (*n *= 10), and normal-saline-only group (negative control, *n *= 5). Bev was injected 1 h before LPS injection. The survival rate of the mice was monitored for seven days after surgery in the CLP mouse model or following LPS injection in the endotoxemia model.

To determine the impact of the time of Bev administration on survival in the murine CLP and endotoxemia models, the mice assigned to each sepsis model were divided into four groups. For the CLP model, the four groups consisted of a CLP control group (*n *= 8) and three groups treated with 0.1 mg/kg Bev i.p. 1 h before CLP surgery, 6 h after CLP surgery or 12 h after CLP surgery; *n *= 8, respectively, denoted as the pre-treated CLP, post-treated CLP 1 or post-treated CLP 2 group. In the endotoxemia model, the four groups consisted of an LPS control group (*n *= 8) and three groups treated with 0.1 mg/kg Bev i.p. 1 h before LPS injection, 6 h after LPS injection or 12 h after LPS injection; *n *= 8, respectively, denoted as the pre-treated LPS, post-treated LPS 1 or post-treated LPS 2 group. The mice were assessed for survival up to seven days following intervention. Mortality rates were compared among groups.

### Permeability assay

The mice were divided into three groups (*n *= 8, respectively). The control group was treated with normal saline, the LPS group was treated with 22 mg/kg LPS-only and the LPS + Bev group was treated with 22 mg/kg LPS and 0.1 mg/kg Bev. Normal saline or Bev was injected 1 h before LPS administration. Twenty-four hours later, mice were anesthetized by i.p. injection of 0.5 ml avertin. Next, 100 μl of 1% Evans blue dye in phosphate-buffered saline (PBS) was injected into the tail vein. Via heart puncture 40 minutes later, mice were perfused with PBS + 2 mM EDTA for 20 minutes. The liver, lung, kidney and spleen were harvested and incubated in formamide for three days to elute the Evans blue dye. The optical density (OD) at 620 nm of the formamide solution was measured [17].

### Cell culture and assay of cellular viability

Human umbilical vein endothelial cells (HUVECs) were purchased from the ATCC (Manassas, VA, USA). Cells were grown according to the ATCC recommendation in F-12K Medium (ATCC), consisting of endothelial cell growth supplement (ECGS, Sigma-Aldrich), heparin (Sigma-Aldrich) and 10% fetal bovine serum (FBS) (Gibco, Gaithersburg, MD, USA). Cells were cultured at 37°C in 5% CO_2_. Cells between passages four and eight were used for all experiments. A cell counting kit-8 (CCK-8) (Dojindo, Rockville, MD, USA) assay was used to assess cell viability.

### Semi-quantitative RT-PCR for measurement of VEGF and cytokine expression levels

HUVECs were harvested from culture dishes and seeded in 60 mm dishes at a density of 1,500 cells/well. The cells were treated with or without Bev 1 h before LPS treatment. Then, the cells were harvested at 1.5 h, 3 h and 5 h after LPS treatment (*n *= 4, per each time). We checked the expression of mRNA of cytokines at each time point in duplo and, thereby, determined the point of greatest mRNA expression. Thus, cells were harvested 3 h after LPS treatment and VEGF, IL-6, monocyte chemotactic protein-1(MCP-1), and regulated on activation, normal T-cell expressed and secreted (RANTES) mRNA levels were determined using a semi-quantitative reverse transcription-polymerase chain reaction (RT-PCR) assay. Briefly, total RNA from HUVECs was isolated using the Easy-spin total RNA extraction kit (iNtRON, Sungnam, Korea) following the manufacturer's instructions. One microgram of total RNA was reverse transcribed using AccuPower Cycle Script RT PreMix (Bioneer, Seoul, Korea), and the cDNA product was amplified with the i-Taq polymerase (iNtRON, Sungnam, Korea) using VEGF-specific primers (forward, 5'-GGTGAGAGGTCTAGTTCCCGA-3'; reverse, 5'-CCATGAACTTTCTGCTCTCTTG-3'), IL-6-specific primers (forward, 5'-CCACAAGCGCCTTCGGTCCA-3'; reverse, 5'-GGGCTGAGATGCCGTCGAGGA-3'), MCP-1-specific primers (forward, 5'-TTCTGTGCCTGCTGCTCATA-3'; reverse, 5' CAGATCTCCTTGGCCACAAT-3'), RANTES-specific primers (forward, 5'-ACAGGTACCATGAAGGTCTC-3'; reverse, 5'-TCCTAGCTCATCTCCAAAGA-3'), or glyceraldehyde 3-phosphate dehydrogenase (GAPDH)-specific primers (forward, 5'-GTCAGTGGTGGACCTGACCT-3'; reverse, 5'-TGAGCTTGACAAAGTGGTCG-3'). The results were quantified using the Multi-gauge ver 3.0 software (Fujifilm, Tokyo, Japan).

### Cytokine measurement in HUVECs using ELISA

HUVECs were cultured to 60 to 70% confluency. Media were removed at 24 h and stored at -70°C until analysis. Levels of IL-6, MCP-1 and RANTES in HUVEC culture supernatants were determined using Quantikine enzyme-linked immunosorbent assay (ELISA) kits. Mouse IL-6 and MCP-1 ELISA kits were purchased from BD→ BD Biosciences (San Diego, CA, USA), mouse RANTES ELISA kits were purchased from R&D Systems (Minneapolis, MN, USA). All ELISA kits were used according to the manufacturer's recommendations.

### Statistical analyses

The non-parametric Mann-Whitney test was used to compare groups. Multiple differences among groups were evaluated using one-way ANOVA multiple comparison test. Survival analyses were performed using Kaplan-Meier curves and the log-rank test. The numeric data presented are the means ± standard deviation. Statistical significance was set at *P *<0.05. SPSS for Windows, version 18.0 (SPSS Inc., Chicago, IL, USA) were used for these analyses. In addition, statistical power was calculated using PASS 2008 (Power Analysis and Sample Size; NCSS, Kaysville, UT, USA) software.

## Results

### VEGF mRNA expression in HUVECs

**Figure 1:**
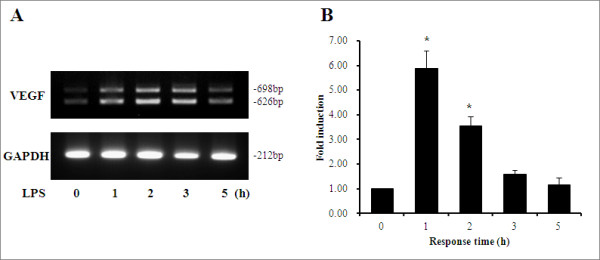
**The expression of VEGF mRNA in HUVECs**. Human umbilical vein endothelial cells (HUVECs) were stimulated with 1 μg/ml lipopolysaccharide (LPS) for up to 5 h. Vascular endothelial growth factor (VEGF) and glyceraldehyde 3-phosphate dehydrogenase (GAPDH) mRNA levels were semi-quantified by RT-PCR. Error bars represent SD. *P <0.01 compared to non-treated HUVECs (*n *= 4).

The time course of LPS-induced expression of VEGF mRNA was assessed initially. VEGF mRNA levels peaked after 1 to 2 h of LPS stimulation (Figure 1A, B). Thus, LPS is a potent mediator of VEGF gene expression in HUVECs.

### Bev inhibits the expression and decreases the concentration of LPS-induced cytokines in HUVECs

**Figure 2:**
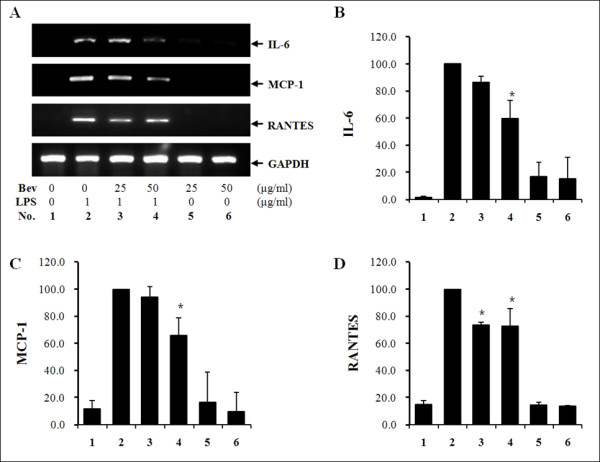
**Expression of IL-6, MCP-1 and RANTES in HUVECs after treatment with LPS and/or bevacizumab**. **A**, cDNA of cytokines and glyceraldehyde 3-phosphate dehydrogenase (GAPDH). **B**, semi-quantitative IL-6 levels. **C**, semi-quantitative monocyte chemotactic protein-1 (MCP-1) levels. **D**, semi-quantitative RANTES (regulated on activation, normal T-cell expressed and secreted) levels. Error bars represent SD. *P <0.01 when compared to lipopolysaccharide (LPS)-only treated human umbilical vein endothelial cells (HUVECs) (*n *= 4).

Next, the levels of IL-6, MCP-1 and RANTES mRNAs in HUVECs were determined (Figure 2). Cytokine levels in cells treated with LPS and Bev were significantly changed when compared to cells treated with LPS alone. For IL-6 and MCP-1, *P *<0.01 was set at 50 μg/ml Bev; for RANTES, *P *<0.01 at both 25 and 50 μg/ml Bev, respectively. Cytokine levels of in cell culture supernatant were also assessed by ELISA (Table 1). IL-6 levels were significantly lower in LPS + Bev (both 25 and 50 μg/ml) treated groups than in the LPS-only group (*P *<0.01). In addition, MCP-1 and RANTES levels were significantly decreased when LPS was administered with 50 μg/ml Bev compared to the LPS-only group.

**Table 1:** The concentration of cytokines IL-6, MCP-1 and RANTES in each group

Cytokines	Control	LPS	LPS + Bev 1	LPS + Bev 2	Bev
**IL-6 (pg/ml)**	0.0 ± 0.0	2,054.3 ± 98.6	1,477.8 ± 44.6^*^	1,281.4 ± 25.9^*^	0.0 ± 0.0
**MCP-1 (ng/ml)**	1.42 ± 0.02	13.71 ± 0.18	10.74 ± 0.25	10.05 ± 0.13^*^	0.87 ± 0.02
**RANTES (pg/ml)**	0.0 ± 0.0	286.2 ± 4.0	240.6 ± 3.3	201.7 ± 17.2^*^	0.0 ± 0.0

Data are expressed as means ± SD. The levels of IL-6, MCP-1 and RANTES in HUVECs after treatment with LPS (LPS group), LPS + Bev (Bev 1 at 25 μg/ml Bev, and Bev 2 at 50 μg/ml Bev), or Bev-only group (50 μg/ml Bev). ^*^P <0.01 when compared to the group of LPS (*n *= 4).

Bev, bevacizumab; HUVECs, human umbilical vein endothelial cells; LPS, lipopolysaccharide; MCP-1, monocyte chemotactic protein-1; RANTES, regulated on activation, normal T-cell expressed and secreted; SD, standard deviation.

### Effect of Bev dose on mortality

**Figure 3:**
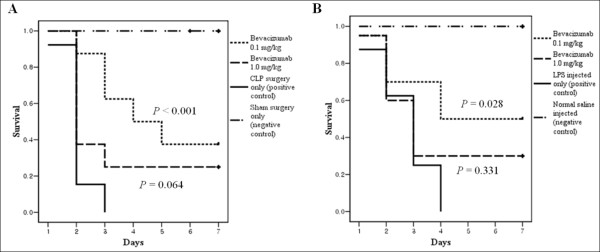
**Survival in murine sepsis models and the effects of differing doses of bevacizumab on mortality**. **A**, Kaplan-Meier survival analysis following cecal ligation and puncture (CLP) comparing bevacizumab (Bev)-treated animals administered 0.1 mg/kg (*n *= 8) or 1.0 mg/kg (*n *= 8) i.p. 1 h before CLP to controls with CLP (*n *= 13). The group administered 0.1 mg/kg had a significantly greater survival than the CLP controls (*P *<0.001). **B**, Kaplan-Meier survival analysis following lipopolysaccharide (LPS) injection comparing Bev-treated animals administered 0.1 mg/kg (*n *= 10) or 1.0 mg/kg (*n *= 10) i.p. 1 h before LPS treatment to mice administered LPS-only (*n *= 8). Administration of 0.1 mg/kg led to significantly greater survival relative to the LPS-only group (*P *= 0.028).

All mice in the control groups (sham surgery and non-LPS treated groups) remained healthy and survived up to seven days, whereas all mice in the CLP only group died within three days of surgery. In contrast, at the seventh experimental day in the CLP with 0.1 and 1.0 mg/kg Bev, groups three and two mice, respectively, were alive. Figure 3A shows the survival curve for each of the four groups. In the LPS-induced endotoxemia model, a similar situation was observed: in the LPS and 0.1 mg/kg Bev group, the mortality rate was significantly reduced, but not in the LPS and 1.0 mg/kg Bev group (*P *= 0.028) (Figure 3B).

### Effect of timing of Bev treatment on mortality in the CLP and endotoxemia models

**Figure 4:**
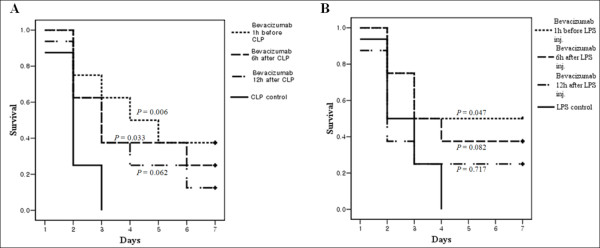
**Effects of differing bevacizumab treatment times on mortality in the murine models of sepsis**. **A**, Delayed administration of Bev is protective in cecal ligation and puncture (CLP). Kaplan-Meier survival analysis following CLP in mice comparing the efficacy of pre- and post-surgical bevacizumab (Bev) treatment at various time intervals relative to the CLP control. Bev administration significantly enhanced survival relative to the CLP controls (*P *= 0.006 in the pre-treated group, and *P *= 0.033 in the 6 h post-treated group), except for mice in which Bev administration was delayed for 12 h (12 h delayed-treatment group, *P *= 0.062). **B**, Lethality from endotoxemia was diminished with delayed Bev administration, but not statistically significantly so.

The effects of delayed administration of 0.1 mg/kg Bev are shown in Figure 3. The administration of Bev provided significant protection up to 6 h after CLP (*P *= 0.033, Figure 4A). In the endotoxemia model, delaying Bev administration until 6 h after LPS treatment also showed a favorable survival trend, but the differences were no longer significant compared with the LPS-only group (*P *= 0.082, Figure 4B).

### Effect of Bev on vascular permeability

**Figure 5:**
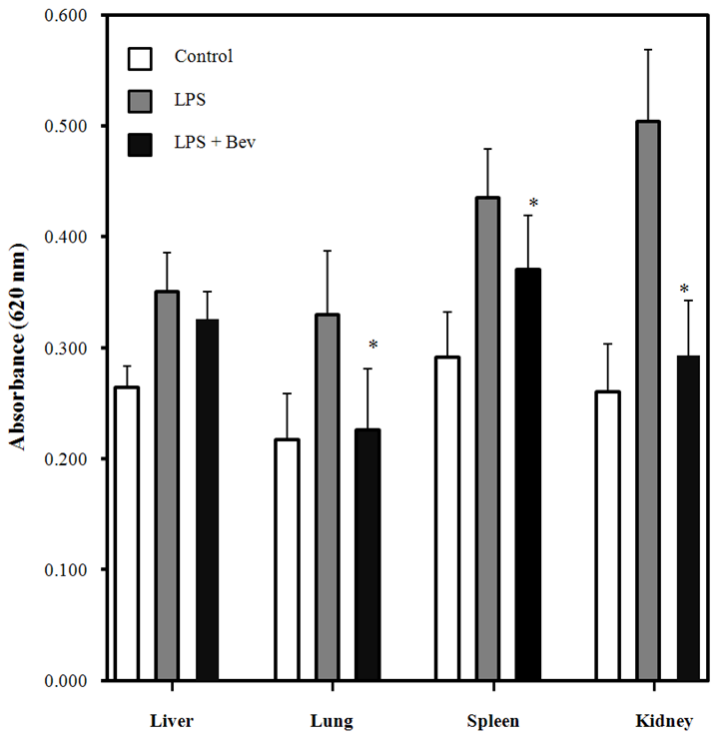
**Effect of bevacizumab treatment on vascular permeability in a mouse model of endotoxemia**. Quantitation of Evans-blue extravasation (optical density (OD) at 620 nm). Error bars represent SD. *P <0.05 when compared to the LPS group (*P *= 0.011 for lung, *P *= 0.046 for spleen, and *P *<0.001 for kidney).

LPS administration resulted in organ-specific loss of barrier function, with increased extravasation of Evans blue dye in the liver, lung, spleen and kidneys (Figure 5). Bev-treatment significantly inhibited vascular permeability in the lung, spleen and renal parenchyma in the mouse model of endotoxemia.

## Discussion

VEGF, an endothelial growth factor widely known for its role in the regulation of embryonic and post-natal angiogenesis, was first characterized as a potent stimulator of endothelial permeability due to its endothelial barrier-breaking properties [4]. The clinical relevance of this effect in humans was reported more than a decade ago, when patients were treated with low doses of VEGF to boost revascularization in critical limb ischemia. Peripheral edema was a recurrent adverse event [18]. Indeed, elevated VEGF levels are associated with conditions that disrupt the endothelial barrier, including sepsis [19,20]. Moreover, levels of VEGF in intensive care patients with sepsis are associated with disease severity and mortality [11,12]. In these studies, we demonstrate that an anti-VEGF antibody protects mice from the lethality of severe peritonitis and endotoxemia. Thus, inhibition of VEGF activity may contribute to sepsis treatment in the future.

Severe sepsis is a major clinical problem in acute care medicine and surgery, yet treatment options remain limited [21,22]. Since severe sepsis is associated with an unacceptably high mortality rate, an important goal is to identify novel therapeutic targets. However, further advances in therapy will be critically dependent on an improved understanding of sepsis pathophysiology. In response to a pathogen, large quantities of proinflammatory cytokines are released in an unregulated immune cascade that can cause multiple organ failure [23,24]. Alterations in the microcirculation may play a critical role in the pathophysiology of sepsis [25]. LPS is one of the most potent microbial mediators implicated in the septic response. LPS triggers proinflammatory cytokine production, and also disrupts the microcirculation by increasing vascular permeability in experimental models [26-28]. In our study, we demonstrate that Bev attenuates excessive vascular permeability in endotoxemia models, and is able to significantly quell the LPS-induced inflammation in HUVECs. Soluble Flt (sFlt)-1, a splice variant of the VEGF receptor VEGFR-1, is secreted, binds VEGF and acts as a decoy receptor, decreasing its net activity. In a previous study, sFlt-1 was shown to protect mice from VEGF-induced sepsis [29] and could play an important role in the treatment of sepsis. In addition, Yano *et al*. showed that adenovirus mediated over-expression of sFlt-1 blocked endotoxemia induced vascular permeability and mortality in mice, and protected against cardiac dysfunction and mortality in a CLP model [17]. In contrast, Nolan *et al*. reported that blocking of VEGF using VEGF trap (VEGF_T_) did not alter lung leakage or mortality but reduced production of IL-6 and IL-10 [30]. VEGF_T _is a recombinant protein generated by the fusion of two domains of VEGFR-1 and 2 attached to the hinge region of the Fc portion of IgG1. VEGF_T _was rationally designed as an extremely high-affinity trap for VEGF and other VEGFR ligands. Whether the benefit of VEGF_T _and Bev parallel that of sFlt-1 is not clear. Differences in affinity for VEGF could affect survival benefit and vascular permeability in animal models of sepsis.

How VEGF functions in sepsis is not completely understood. However, significant insights regarding plasma and pulmonary VEGF in sepsis have been garnered from animal models. Kaner *et al*. demonstrated that intrapulmonary over-expression of VEGF results in high-permeability edema in the lungs of mice [31], which was blocked by a biological inhibitor of VEGF. This suggests that VEGF regulates baseline microvascular permeability and that elevated alveolar VEGF levels might determine pulmonary edema in acute respiratory distress syndrome [31]. Therefore, ability of Bev to neutralize VEGF, -like sFlt-1, may attenuate morbidity and mortality in severe sepsis.

In our study, survival benefits were evident in mice given 0.1 mg/kg rather than 1.0 mg/kg Bev. Bev inhibits the growth of human tumor cell lines in nude mice, achieving a maximal inhibition at the dose of 1 to 2 mg/kg twice per week [32]. Half-maximal inhibition required 0.1 to 0.5 mg/kg doses. Why the lower dose of Bev seemed more effective than the higher dose in this study is unclear, although there are three possible explanations: the unique role of Bev as an inhibitor of angiogenesis and wound healing may account for this inverted dose-effectiveness. There are few pre-clinical reports of the effect of the anti-VEGF antibody on wound healing. Bev reduces the rate of spontaneous wound healing in macaques [33]. Also, agents targeting VEGF have deleterious effects on the healing of ventral hernias and colonic anastomoses [34-36]. In humans, dose-limiting toxicity was induced in locally advanced rectal cancer patients [37]. Wound-healing complications might be possible causes for different survival benefits that are dose-dependent. However, the finding in endotoxemia models has not been fully explained. Reduction in intra-tumor pressure and improved delivery of chemotherapy result in greater Bev efficacy than induction of vascular collapse inside the tumor [38,39]. The lower dose may have resulted in improved delivery of antibiotics or leukocytes to clear the infection, whereas the higher dose resulted in vascular collapse, limiting delivery of antibiotics or leukocytes. Bev was generally well tolerated in clinical studies, and a recent study showed that the plasma VEGF level increases during the first 48 h of human septic shock and correlates with vascular permeability [11]. Therefore, the impact of Bev on severe sepsis should be confirmed by further research including determination of tissue-specific toxicities.

The current study has some limitations that need to be addressed. First, the different sizes of groups of mice were used in survival analyses. However, a power calculation found that high and even statistical power could be confirmed. Secondly, the mechanisms of the inverted dose response were not clearly evaluated. Furthermore, the data presented do not support the use of Bev for the clinical treatment of sepsis. Therefore, further studies are needed to evaluate the optimal dosage, safety, efficacy and other effects of Bev for sepsis.

## Conclusions

This is, to our knowledge, the first report to document the therapeutic effects of Bev in two standard murine models of sepsis: polymicrobial sepsis resulting from a ruptured viscus and endotoxemic sepsis. The attenuation of VEGF activity by Bev may be an effective approach to the treatment of severe sepsis in clinical settings. Further investigation of the safety and possible synergistic effects in combination with antibiotics will be needed to determine the feasibility of using this strategy for treatment of life-threatening severe sepsis.

## Key messages

• Bev administration significantly decreased mRNA and protein levels of the pro-inflammatory cytokines IL-6, MCP-1 and RANTES in HUVECs treated with LPS when compared to LPS-only treated HUVECs.

• A dose of 0.1 mg/kg increased survival relative to the CLP control and LPS control groups in both the murine CLP and endotoxemia models of sepsis.

• Bev administration provides significant protection up to 6 h after CLP, but the differences were no longer significant compared with the LPS control group.

• Bev-treatment inhibited vascular permeability in the lung, spleen and renal parenchyma in a mouse model of endotoxemia.

## Abbreviations

Bev: bevacizumab; CCK-8: cell counting kit-8; CLP: cecal ligation and puncture; CX3CL1: C-X3-C motif ligand 1; FBS: fetal bovine serum; HUVECs: human umbilical vein endothelial cells; ICAM-1: intercellular adhesion molecule 1; IL-β: interleukin-1β; IL-6: interleukin-6; i.p.: intraperitoneal; LPS: lipopolysaccharide; MCP-1: monocyte chemotactic protein-1; OD: optical density; PBS: phosphate-buffered solution; PIGF: placental growth factor; RANTES: regulated on activation: normal T-cell expressed and secreted; rhAPC: recombinant activated protein C; TLR: toll-like receptor; TNF-α: tumor necrosis factor-α; VCAM-1: vascular cell adhesion molecule 1; VEGF: vascular endothelial growth factor; VEGFR: vascular endothelial growth factor receptor

## Competing interests

The authors declare that they have no competing interests.

## Authors' contributions

SJJ, COK, JYC and JMK were responsible for study conception and design. SJJ, SHH and JYC acquired the data and conducted the statistical analysis and interpretation of data. SJJ and SHH drafted the manuscript. SHH, COK, JYC and JMK conducted a critical revision of the manuscript for important intellectual content. All authors have read and approved the manuscript for publication.
